# Comparative Analysis of Intense Pulsed Light Therapy in Patients with Meibomian Gland Dysfunction, with and Without Glaucoma Medication

**DOI:** 10.3390/jcm13216341

**Published:** 2024-10-23

**Authors:** Kwang-Eon Han, Jinho Kim, Su-Jin Kim, Jonghoon Shin, Eun-Jung Choi, Sangmin Kim, Dogyu Lee, Jeongyun Kim, Sangwoo Moon, Ji-Eun Lee

**Affiliations:** 1 Department of Ophthalmology, Pusan National University Yangsan Hospital, Pusan National University School of Medicine, Yangsan 43241, Republic of Korea; kehanoph@pusan.ac.kr (K.-E.H.); pearlsj@hanmail.net (S.-J.K.); 2 Research Institute for Convergence of Biomedical Science and Technology, Pusan National University Yangsan Hospital, Pusan National University School of Medicine, Yangsan 43241, Republic of Korea; 3 Department of Medicine, Pusan National University School of Medicine, Yangsan 43241, Republic of Korea; dandobby5213@pusan.ac.kr (J.K.); osfmin@pusan.ac.kr (S.K.); ldg9088@pusan.ac.kr (D.L.); kjy5376@pusan.ac.kr (J.K.); 4 PNU Busan Eye Clinic, Busan 46241, Republic of Korea; ophdrshin@gmail.com; 5 Bio-IT Fusion Technology Research Institute, Pusan National University, Busan 46241, Republic of Korea; eunjung721203@gmail.com

**Keywords:** dry eye disease, meibomian gland dysfunction, glaucoma, intense pulsed light

## Abstract

**Background**: This study assessed the efficacy of intense pulsed light (IPL) therapy for treating meibomian gland dysfunction (MGD), a key contributor to evaporative dry eye disease (DED), by comparing outcomes in patients with idiopathic MGD versus those with MGD induced by glaucoma medications. **Methods**: In a retrospective analysis of 45 patients, divided into groups based on glaucoma medication use (20 patients) and non-use (25 patients), all underwent four IPL sessions combined with meibomian gland expression (MGX) at 3-week intervals. Key metrics evaluated included Ocular Surface Disease Index (OSDI) scores, tear breakup time (TBUT), Schirmer I test scores, and meibography scores, pre- and post-treatment. **Results**: Significant improvements were observed in both groups across all parameters post-treatment, indicating enhanced tear film stability and meibomian gland function. The non-glaucoma group showed slightly greater improvements, suggesting the potential impact of glaucoma medications on MGD management. **Conclusions**: These findings underscore IPL therapy’s effectiveness in improving DED symptoms and meibomian gland function, highlighting its utility as a treatment option for patients with MGD, including those on glaucoma medications.

## 1. Introduction

Dry eye disease (DED) is a common condition, with meibomian gland dysfunction (MGD) being a major contributor to evaporative DED [1]. Among these, meibomian gland dysfunction (MGD) emerges as a prominent contributor and is notably prevalent in the increased evaporation type of DED [2,3]. MGD, characterized by a dysfunction of the meibomian glands responsible for producing the lipid layer of the tear film, has gained significant attention in recent years due to its pivotal role in DED pathophysiology. Consequently, the pursuit of effective MGD treatments has become a focal point in ocular research.

In response to the growing understanding of the impact of MGD on DED, numerous studies have been conducted over the past decade. Recognizing MGD as a base of DED, researchers have dedicated their efforts to exploring innovative and efficient therapeutic interventions, including promising approaches such as intense pulsed light (IPL) therapy, which has shown remarkable effectiveness in managing MGD in general DED cases. IPL has been well-established as an effective treatment for managing MGD by reducing inflammation and improving meibomian gland function. [4]. This improves gland function by obstructing abnormal vessels from secreting inflammatory mediators [5]. IPL therapy has expanded its application beyond its initial scope and is now being explored in various ocular conditions including contact lens-related dry eye [6], DED after corneal refractive surgery [7], recurrent chalazia [8], and glaucoma [9].

However, limited research has been conducted on the therapeutic effect of treating DED due to glaucoma eye drops with IPL [9]. The number of patients with glaucoma is expected to be 111.8 million worldwide in 2040 [10]. Prostaglandin analogs, beta-adrenergic antagonists, alpha-adrenergic agonists, and topical carbonic anhydrase inhibitors are commonly used to manage glaucoma topically [11]. However, these treatments can potentially cause or worsen ocular surface disease, either due to the preservatives or the active ingredients in the medication [11]. IPL therapy combined with meibomian gland expression (MGX) has shown promise as an effective treatment for ocular surface disease resulting from frequent use of hypotensive eyedrop in patients with glaucoma [9].

To the best of our knowledge, this is the first study that compares the therapeutic effects of IPL treatment in idiopathic MGD and topical glaucoma medication-induced MGD. Due to the differences in the mechanism between the MGD caused by glaucoma eye drops and MGD without glaucoma eye drops, it is important to evaluate whether IPL treatments show similar or different efficacy in these two comparative patient groups. This study aimed to evaluate the efficacy of IPL for MGD caused by glaucoma eye drops and compare the therapeutic effects of IPL for MGD resulting from glaucoma eye drops and MGD without glaucoma eye drops.

## 2. Materials and Methods

### 2.1. Patients

This study received ethical approval from the Institutional Review Board (IRB) of Pusan National Yangsan Hospital, Pusan, Republic of Korea (IRB approval number: 05-2023-148) and was conducted in accordance with the principles of the Declaration of Helsinki. The criteria for inclusion encompassed individuals aged between 18–85 years who satisfied all of the subsequent conditions: an Ocular Surface Disease Index (OSDI) score of 13 points or higher; a tear breakup time (TBUT) of <10 s for both eyes [12]; and the presence of at least two clinical indicators associated with MGD, such as eyelid margin redness or thickening, telangiectasia, diminished or absent secretions, secretion of poor quality, and meibomian gland capping [13]. The criteria for exclusion included individuals with Sjögren’s syndrome, a history of prior intraocular or ocular surgical procedures, eyelid malposition, current ocular infection, non-dry eye-related ocular inflammation, ocular allergies, any history of autoimmune disorders, use of contact lenses during the research period, undergoing clinical skin treatments within the 3 months preceding the study, having semi-permanent makeup or tattoos, the presence of pigmented lesions at the treatment area, and those who were pregnant or lactating. One patient was excluded due to Sjögren’s syndrome, and among the remaining four, three had a history of prior ocular surgical procedures, and one was excluded due to a suspected ocular infection. From the pool of patients who met the above criteria, we selected two groups: 20 patients with MGD who were concurrently using glaucoma eye drops and undergoing continuous IPL therapy, and another 25 patients with MGD who were receiving the same continuous IPL therapy without the use of glaucoma eye drops. All patients using glaucoma eye drops administered the drops in both eyes. All participants underwent an identical IPL therapy protocol, which was administered by a single trained physician (J.E.L.).

### 2.2. Clinical Assessment

**Figure 1:**
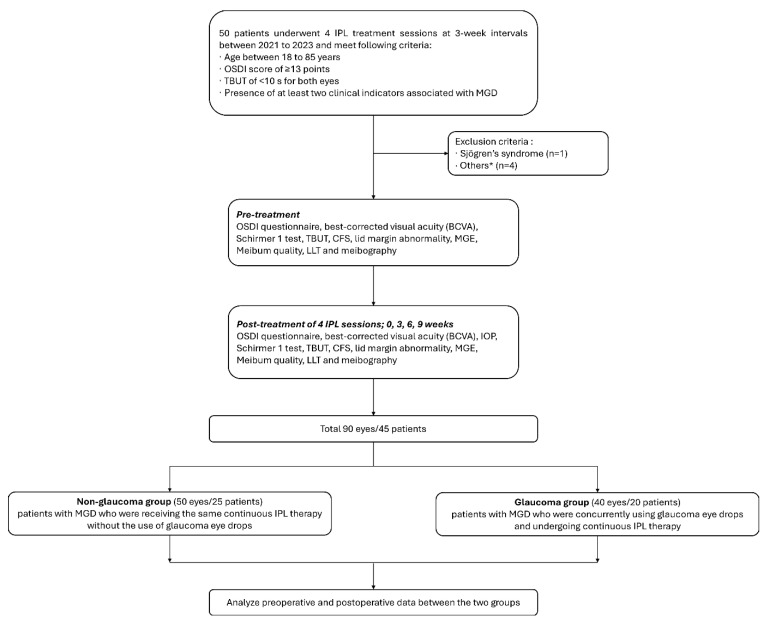
Subject disposition and study protocol are described with a flow chart. * Others: eyelid malposition, current ocular infection, non-dry eye-related ocular inflammation, ocular allergies, any autoimmune disorders, use of contact lenses during the research period, undergoing clinical skin treatments within the three months preceding the study, having semi-permanent makeup or tattoos, the presence of pigmented lesions at the treatment area, pregnancy or lactating.

Before deciding on IPL treatment, routine evaluations were conducted to assess the effectiveness of the procedure, including the OSDI questionnaire, best-corrected visual acuity (BCVA), the 5-min Schirmer 1 test, TBUT, corneal fluorescein staining score (CFS), lid margin abnormality, meibomian gland expressibility (MGE), meibum quality, lipid layer thickness (LLT) measurement, and meibography. To estimate the degree of patients’ discomfort, participants filled out the OSDI questionnaire comprising 12 questions before the IPL treatment [14]. BCVA was assessed along the standardized protocol that follows manifest refraction assessment in both eyes. A Snellen chart in a standard light box was 3 m away from the patient. BCVA results were modified to the logarithm of the minimum angle of resolution (logMAR) to proceed with statistical analysis. The Schirmer I test was conducted using sterile Schirmer strips (Eagle Vision, Memphis, TN, USA), without anesthesia. Strips were placed in the middle of the lower fornix for 5 min. The length of the tape’s wet section was measured in millimeters, and the test was considered positive if the length of the wet paper was less than 5 mm [15]. TBUT, CFS, and lid margin abnormality assessments were conducted using slit lamp examination, while LLT measurement and meibography were performed with the LipiView ocular surface interferometer (TearScience Inc., Morrisville, NC, USA). MGE and meibum quality were examined before and after the IPL treatment sessions, whereas the remaining assessments were conducted before IPL therapy. In addition to the routine evaluations and clinical assessments conducted prior to IPL treatment, all procedures, including the assessment of MGE and meibum quality, were performed by a single, trained physician (J.E.L.) to ensure consistency and minimize variability in the evaluation process. Lid margin abnormalities were assessed according to the following four signs [16]: lid margin irregularity, vascular engorgement, plugged meibomian gland orifices, and displacement of the mucocutaneous junction. MGE was graded as follows: 1, all glands; 2, 3–4 glands; 3, 1–2 glands; and 4, no gland expressible. Meibomian quality was graded as follows: grade 1, clear fluid; 2, cloudy liquid secretion; 3, particulate; and 4, inspissated [17]. The meiboscore classification categorizes the extent of meibomian gland loss in both the upper and lower eyelids into four distinct grades: grade 0, no meibomian gland loss; 1, loss of less than one-third of the meibomian glands’ total area; 2, glandular loss ranging from one-third to two-thirds of the total area; and 3, loss of more than two-thirds of the meibomian glands’ surface area [3,18,19]. Four weeks after the final IPL session, patients were re-evaluated using the identical tests and examinations conducted before the IPL treatment. Data on the same variables, along with any adverse effects, were systematically documented (Figure 1).

### 2.3. IPL Treatment Protocol

A single ophthalmologist utilized the Lumenis M22 (M22: Luminism Ltd., Yokneam, Israel) throughout the study. They both had four IPL treatment sessions at 3-week intervals, treating both eyes on the same day, starting with the right eye. This therapeutic approach adhered to a methodology outlined in the prior literature [18] The therapeutic process was as follows: initially, topical 0.5% proparacaine anesthetic eye drops (Paracaine; Hanmi Pharm, Seoul, Republic of Korea) were applied to each eye. Subsequently, a thin layer (approximately 1 mm) of cooled ultrasound gel was coated over the eyelids area. To protect the eye, the Jaeger lead plate (Katena Products, Denville, NJ, USA) was placed on the conjunctival sac. The actual IPL treatment involved delivering a series of 20 overlapping pulses to the skin, applied from the right temple, across the lower eyelid, including the bridge of the nose, and extending to the left temple along the lower eyelid. The same procedure was performed for the upper eyelid. This process was repeated twice, utilizing a 590-nm filter with a 6 mm cylindrical light guide. Following the IPL treatment, MGX was immediately operated on both the upper and lower eyelids with eyelid compression forceps (Katena Products, Parsippany, NJ, USA).

### 2.4. Statistical Analysis

Each eye was considered an individual data point for statistical analysis. Descriptive statistics were presented as means and standard deviations. The two groups were compared using a Pearson’s chi-square test and a Student’s *t*-test. The normality of data was evaluated using the Shapiro–Wilk test. Statistical differences between baseline scores and scores at each week were evaluated using the analysis of covariance and Wilcoxon signed-rank test. A *p*-value of <0.05 was considered statistically significant (*p* < 0.05). All data were analyzed using SPSS Statistics for Windows (SPSS Version 27.0, IBM Corporation, Armonk, NY, USA).

## 3. Results

This retrospective study included 90 eyes from 45 patients with MGD, each receiving four IPL treatments and MGX sessions at 3-week intervals. Among the participants, 20 were in the glaucoma group and 25 were in the non-glaucoma group, consisting of 8 males and 12 females, and 9 males and 16 females, respectively. The average age was 60.8 ± 7.85 years in the glaucoma group and 58.3 ± 11.4 years in the non-glaucoma group. Pre-treatment BCVA, OSDI, and dry eye parameters showed no significant difference between the two groups (Table 1).

**Figure 2:**
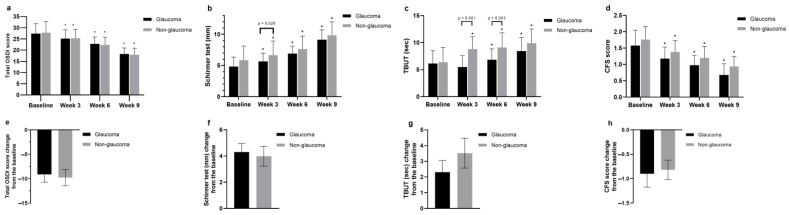
Changes in clinical parameters of dry eye after IPL and MGX treatments. (**a**) OSDI scores showed significant improvement in both the glaucoma and non-glaucoma groups after four treatments, with no significant difference between the groups. (**b**) Schirmer I test results indicated significant improvement in both groups, with a significant difference after the first treatment. (**c**) TBUT improved significantly in both groups, with differences observed after the first and second treatments. (**d**) CFS scores improved significantly in both groups following the treatments. (**e**–**h**) Average changes from baseline in OSDI, Schirmer I, TBUT, and CFS scores showed significant improvement across all parameters. * *p* < 0.05, compared with baseline values by analysis of covariance.

In the glaucoma group, the OSDI scores improved significantly from 27.4 ± 4.44 before treatment to 18.3 ± 2.76 after four treatments. In the non-glaucoma group, they improved from 27.8 ± 5.02 before treatment to 18.0 ± 2.95 following four treatments. Although both groups showed significant improvement (*p* < 0.001 for both groups) after four treatments, no significant difference was observed between the two groups (Figure 2a). In the Schirmer I test, the glaucoma group showed a significant improvement (*p* < 0.001 for both groups) from 4.83 ± 1.52 before treatment to 5.65 ± 1.29 after one treatment and 9.13 ± 1.57 after four treatments. Similarly, the non-glaucoma group exhibited improvement from 5.86 ± 3.04 before treatment to 6.66 ± 2.79 after one treatment and 9.84 ± 2.17 after following treatments. Both groups demonstrated statistically significant improvement after four treatments, with a significant difference between the two groups after the first treatment (*p* = 0.026), suggesting an improvement trend (Figure 2b).

**Figure 3:**
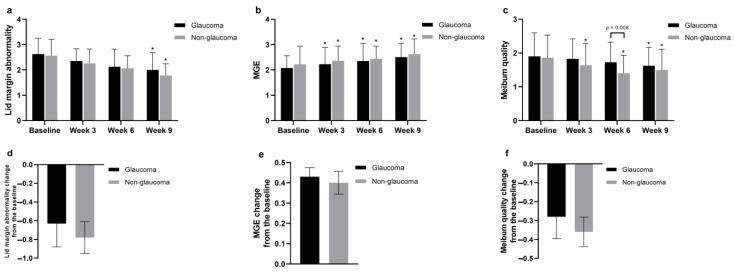
Changes in meibomian gland involvement scores after IPL and MGX treatments. (**a**) Lid margin abnormality scores showed significant improvement in both groups after four treatments. (**b**) MGE scores demonstrated significant improvement in both the glaucoma and non-glaucoma groups. (**c**) Meibum quality improved significantly in both groups. (**d**–**f**) Average changes from baseline in lid margin abnormality, MGE, and meibum quality showed significant improvement across all measures. * *p* < 0.05, compared with baseline values by analysis of covariance.

In the TBUT measurements performed with a slit lamp microscope, the glaucoma group exhibited scores of 6.13 ± 3.47 before treatment, 5.49 ± 3.13 after one treatment, 6.85 ± 3.07 after two treatments, and 8.43 ± 3.52 after four treatments. In the non-glaucoma group, scores were 6.38 ± 4.72 before treatment, 8.8 ± 4.31 after one treatment, 9.1 ± 3.70 after two treatments, and 9.9 ± 3.69 after four treatments. Both groups demonstrated statistically significant improvement (both *p* < 0.001) after four treatments compared to before treatment. Additionally, significant differences were observed between the two groups after the first and second treatments (*p* < 0.001 and *p* = 0.003, respectively) (Figure 2c). The CFS score in the glaucoma group improved from 1.58 ± 0.96 before treatment to 0.68 ± 0.69 after four treatments, and in the non-glaucoma group from 1.76 ± 1.19 before treatment to 0.94 ± 0.1 after four treatments, showing significant improvement (both *p* < 0.001) after four treatments (Figure 2d). Lid margin abnormality scores improved from 2.63 ± 0.63 before treatment to 2.00 ± 0.78 after four treatments in the glaucoma group, and from 2.56 ± 0.64 before treatment to 1.78 ± 0.46 after four treatments in the non-glaucoma group, with both groups showing statistically significant improvement (both *p* < 0.001) (Figure 3a).

**Figure 4:**
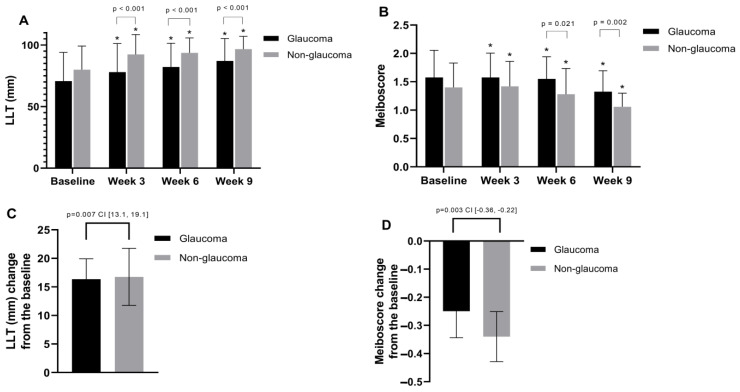
Changes in meibomian gland function parameters after IPL and MGX treatments. (**A**) LLT increased significantly in both groups, with significant differences observed after the second, third, and fourth treatments. (**B**) Meiboscores improved significantly in both groups, with differences between the groups after the third and fourth treatments. (**C**–**D**) Average changes in LLT and meiboscores from baseline showed greater improvement in the non-glaucoma group compared to the glaucoma group. * *p* < 0.05, compared with baseline values by analysis of covariance.

After undergoing IPL and MGX treatments, the evaluation of MGE revealed significant improvement in both groups. In the glaucoma group, the scores improved from 2.08 ± 0.48 before treatment to 2.50 ± 0.55 after four treatments, and in the non-glaucoma group, they improved from 2.22 ± 0.71 before treatment to 2.62 ± 0.60 after four treatments, with both groups exhibiting statistically significant improvement (both *p* < 0.001) (Figure 3b). Regarding meibum quality, in the glaucoma group, scores improved from 1.90 ± 0.48 before treatment to 1.63 ± 0.54 after four treatments, and in the non-glaucoma group, they improved from 1.86 ± 0.67 before treatment to 1.5 ± 0.61 after four treatments. Both groups showed statistically significant improvement after four treatments (*p* = 0.02 and *p* < 0.001, respectively), and a significant difference was observed after the third treatment (*p* = 0.008) (Figure 3c). Post-treatment assessments using LipiView showed improvements in both LLT and meiboscores across both groups. Concerning LLT, in the glaucoma group, the measurements were 70.7 ± 23.3 nm, 77.9 ± 23.2 nm, 82.2 ± 19.3 nm, and 87.1 ± 18.2 nm before, and after two, three, and four treatments, respectively. In the non-glaucoma group, the measurements were 79.9 ± 19.2 before treatment, 92.3 ± 16.1 after two treatments, 93.7 ± 12.0 after three treatments, and 96.7 ± 10.4 after four treatments. Both groups showed statistically significant improvement (both *p* < 0.001) after four treatments compared to before treatment, with significant differences observed after the second, third, and fourth treatments (all *p* < 0.001) (Figure 4A). For the meiboscores, in both groups, scores were 1.58 ± 0.59 for the glaucoma group and 1.40 ± 0.53 for the non-glaucoma group before treatment. After three treatments, the scores were 1.55 ± 0.60 and 1.28 ± 0.45, respectively, and after four treatments, they were 1.33 ± 0.47 and 1.06 ± 0.2, respectively. Both groups showed statistically significant improvement (both *p* < 0.001) after four treatments compared to before treatment, with significant differences observed between the two groups after the third and fourth treatments (*p* = 0.021 and *p* = 0.002, respectively) (Figure 4B).

We conducted an efficacy evaluation by measuring the changes in average scores from baseline after four treatments across all parameters studied (Figure 2e–h, Figure 3d–f, and Figure 4C,D). Statistically significant improvements were observed only in the changes of average LLT and meiboscores, with the non-glaucoma group showing greater enhancement after four treatments. The change in average LLT scores was 16.4 ± 3.58 for the glaucoma group and 16.8 ± 5.00 for the non-glaucoma group. Additionally, the change in average meiboscores was −0.25 ± 0.09 for the glaucoma group and −0.34 ± 0.09 for the non-glaucoma group, indicating that the non-glaucoma group experienced a more significant improvement in these parameters than the glaucoma group following the treatments (Figure 4C,D).

Patients undergoing IPL treatment reported no side effects, such as warmth around the eyes, eyelid swelling, or ocular pain, from immediately after the laser treatment to the completion of the fourth session.

## 4. Discussion

Previous studies have demonstrated IPL’s effectiveness in improving MGD and dry eye symptoms across various patient groups [20,21,22]. Arita et al. [22] compared the changes in meibomian glands in eyes using glaucoma eye drops with their paired eyes not using glaucoma medications and discovered that eyes receiving glaucoma treatments exhibited more significant changes in the meibomian glands. Additionally, they found that the ocular surface test results were poorer in the treated eyes. Nijm et al. [23] studied the prevalence of ocular surface disease in patients with glaucoma, reporting that 40–50% experienced ocular surface diseases. Martinez-de-la-Casa et al. [8] found IPL treatment to be effective in improving ocular surface disease and meibomian function in patients using glaucoma eye drops. Zhang et al. [24] also demonstrated significant improvements in managing glaucoma-related dry eye disease with IPL treatment, highlighting the potential of IPL as a therapeutic option for these patients. Thus, many cases using glaucoma eye drops also present with conjunctival inflammation, damage, and reduced meibomian gland function, and there are reports that IPL and MGX are effective in these patients. However, no study has examined the effectiveness of IPL treatment in individuals with MGD who use glaucoma eye drops compared to those who do not. Therefore, this study aimed to compare changes in ocular surface and tear film indicators, and meibomian gland indicators following IPL treatment between groups using and not using glaucoma eye drops.

OSDI scores were almost similar across both groups, with the trend in score reduction following treatment sessions displaying a similar pattern. Additionally, both groups showed significant improvement after each session, and statistically significant improvement was still observed after four treatment sessions compared to the initial scores. Symptoms of dry eye in patients with MGD are related to signs of meibomian gland dysfunction, especially with a strong correlation between meiboscores and symptoms of dry eye [25,26,27]. In this study, improvements in meiboscores were observed in both groups, suggesting that these improvements in meibomian gland function are associated with improvements in dry eye symptoms. Moreover, improvements exhibited by both groups in OSDI after IPL treatment are consistent with reports evaluating OSDI in each group, indicating enhancements in both [8,28]. The improvements observed in the Schirmer I test, TBUT, CFS scores, and lid margin abnormalities after four treatment sessions, compared to before treatment in both groups, may be attributed to IPL’s capability to enhance the lipid component of the tear layer and alleviate inflammation in the tear glands and eyelids [29,30]. These results are consistent with previous reports that these four tear film, eyelid, and ocular surface indicators improved after IPL treatment for MGD [18,31]. Reports evaluating TBUT, and CFS scores after IPL treatment in patients using glaucoma eye drops also exhibited improvements in these two indicators [8,32]. Although there were no previous reports evaluating the Schirmer I test and lid margin abnormality, the results of this study demonstrated similar improvements after four treatments in the non-glaucoma group, suggesting that IPL’s effect on increasing the lipid component of the tear layer and improving eyelid inflammation was equally effective. The statistically significant improvement in MGE and meibum quality in the non-glaucoma group after four treatments is believed to be facilitated by the heat and infrared radiation emitted by the IPL laser. This aligns with previous studies that have demonstrated the liquefaction of blocked meibum lipids, thereby enhancing secretion from the meibomian glands [33,34]. In the glaucoma group, a significant improvement was also noted, consistent with the findings of Martinez-de-la-Casa et al. [8], where MGE showed significant improvement after treatment. Although there have been no reports evaluating meibum quality after IPL treatment in the glaucoma group to date, the level of improvement observed was similar to that in the non-glaucoma group. This suggests that IPL treatment might have a comparable effect in improving meibum quality across both groups, despite the absence of specific prior research in the glaucoma population. This interpretation indicates the broad applicability of IPL therapy in enhancing meibomian gland function, regardless of glaucoma presence. Significant improvements in LLT and meiboscores were observed in both groups after four treatments, reflecting outcomes reported by other studies that investigated these two indicators following IPL treatment in the MGD group [18,34,35]. Moreover, several reports have indicated a decrease in LLT and meiboscores following the use of glaucoma eye drops, with one study reporting an improvement in meiboscores in the glaucoma group after IPL treatment, which could support our research findings [8,36,37]. This improvement pattern is believed to be due to enhanced meibomian gland secretion, which prevents meibomian gland atrophy and improves damaged gland function [38].

This study showed significant differences between the groups in the Schirmer I test, TBUT, and LLT after the second IPL treatment, and in TBUT, meibum quality, LLT, and meiboscores after the third treatment. However, it is crucial to note that baseline MGD severity did not differ significantly between the groups. This discrepancy may be explained by the adverse effects of glaucoma medications, particularly prostaglandin analogs (PGAs) and preservatives like benzalkonium chloride (BAK). PGAs are known to induce chronic inflammation and promote angiogenesis, leading to damage in the meibomian glands, while BAK can cause direct cytotoxicity to ocular surface cells and exacerbate meibomian gland dysfunction. These factors disrupt tear film stability and contribute to gland atrophy, making functional recovery more challenging. Studies have shown that up to 80% of patients on PGAs experience moderate to severe MGD, compared to 52.5% in the control group without glaucoma treatment [22,39]. This suggests that prolonged use of glaucoma medications, particularly those containing preservatives like benzalkonium chloride (BAK), contributes to more severe gland atrophy, which can delay functional recovery, as observed in our study [40]. Significant differences observed in LLT and meiboscores by the fourth treatment suggest that, unlike other parameters, improvements in the function of the meibomian glands, as indicated by LLT and meiboscores, are known to become apparent only after the structure and atrophy of the meibomian glands have recovered. Additionally, when comparing the average changes in all parameters from baseline to after four treatments, statistically significant differences were observed only in LLT and meiboscores, further highlighting the distinct improvements in these parameters. These outcomes are believed to be due to the nature of LLT and meiboscores, which reflect the functional recovery of the meibomian glands following structural restoration [41]. As Lu et al. [42] highlighted, IPL tends to be more effective with an increased number of sessions. Given that recovery in glaucoma patients tends to be slower, additional IPL treatments could further improve both the ocular surface symptoms and the structure and function of the meibomian glands in these patients. Therefore, if patients are able to manage their time and financial commitments, continuous IPL treatment could be beneficial.

This study is significant for evaluating the effects of IPL and MGX treatment on MGD in patients using glaucoma medication, incorporating assessments of eyelid, tear film, ocular surface indicators, and direct evaluation of meibomian gland function. Additionally, observing structural changes in the meibomian glands through gland imaging and comparing these findings with those not using glaucoma medication who underwent the same tests provides valuable insights.

However, our research had several limitations. First, it is a retrospective cohort study conducted at a single center with a small participant number. Second, in clinical practice, patients who do not respond to IPL treatment may receive additional treatments. Our study only observed outcomes up to the fourth treatment, which makes it challenging to determine whether non-responding patients might improve with further treatment or assess the potential for long-term side effects. Third, potential bias may arise from the influence of previously used dry eye drops for treating dry eye syndrome. Ceasing these drops could raise ethical concerns, thus maintaining this bias. Lastly, it is recognized that the type and number of glaucoma eye drops may variably impact ocular surface anomalies and meibomian gland abnormalities, underscoring the necessity for further comprehensive research considering these factors.

Despite these limitations, within the context of limited research reporting the outcomes of combined IPL and MGX treatment in patients with MGD using glaucoma eye drops, our study has demonstrated the potential for this treatment to contribute to functional and structural improvements of the meibomian glands as well as in the enhancement of eyelid, tear film, and ocular surface indicators. Hence, in patients with MGD who are using glaucoma eye drops, combined IPL and MGX therapy could serve as a safe and effective adjunctive treatment option. However, when comparing the outcomes in terms of changes in parameters between patients with MGD not using glaucoma eye drops and those who do, significant differences were observed in parameters indicative of meibomian gland function, such as LLT and meiboscores, with improvements noted in the non-glaucoma MGD group. Therefore, for patients with MGD on glaucoma eye drops undergoing IPL treatment, it is advisable to pursue a continuous therapy approach without limitations on the number of treatments, along with a comprehensive follow-up strategy. This approach could enhance the treatment’s efficacy and provide better management of their condition.

## Figures and Tables

**Table 1:** Baseline characteristics of two groups.

Parameter	Glaucoma Group (*n* = 20)	Non-Glaucoma Group (*n* = 25)	*p*-Value
Eyes	40	50	*n*/A
Sex (male/female)	8/12	9/16	0.697 ^†^
Age (years)	60.8 ± 7.85	58.3 ± 11.4	0.250 *
BCVA (logMAR)	0.18 ± 0.34	0.12 ± 0.25	0.344 *
OSDI	27.4 ± 4.44	27.8 ± 5.02	0.705 *
Schirmer test (mm)	4.83 ± 1.52	5.22 ± 2.10	0.321 *
TBUT (s)	6.13 ± 3.47	6.38 ± 4.72	0.768 *
CFS score	1.58 ± 0.96	1.76 ± 1.19	0.427 *
Lid margin abnormality	1.85 ± 0.70	2.14 ± 0.78	0.071 *
MGE	2.33 ± 0.73	2.56 ± 0.73	0.134 *
Meibum quality	1.90 ± 0.71	1.86 ± 0.67	0.785 *
LLT (nm)	71.7 ± 22.4	79.9 ± 19.2	0.069 *
Meiboscore	1.58 ± 0.59	1.40 ± 0.54	0.146 *

Values are presented as mean ± standard deviation, number, or number (%) unless otherwise indicated. N/A = not applicable; BCVA = best-corrected visual acuity; logMAR = logarithm of the minimum angle of resolution; OSDI = Ocular Surface Disease Index–a questionnaire that assesses the frequency and severity of dry eye symptoms; TBUT = tear break-up time–a measure of the time taken for the tear film to break up on the ocular surface after a blink, indicating tear film stability; CFS = corneal and conjunctival staining–an assessment of ocular surface damage using fluorescein dye; MGE = meibomian gland expressibility–a measure of how easily the meibomian glands release their contents upon gentle pressure; LLT = lipid layer thickness–an indicator of the thickness of the tear film’s lipid layer, which helps maintain tear film stability. * Comparison between groups by Student *t*-test. ^†^ Comparison between groups by Pearson’s chi-square test.

## Data Availability

All data pertaining to the study was described in the manuscript.
